# Factors associated with acute malnutrition among children aged 6–59 months in Haiti, Burkina Faso and Madagascar: A pooled analysis

**DOI:** 10.1371/journal.pone.0278980

**Published:** 2022-12-12

**Authors:** Ali-Mohamed Nassur, Oussama Daanouni, Gwenaelle Luc, Alexandra Humphreys, Lenka Blanarova, Grace Heymsfield, Firmin Kouassi, Suvi T. Kangas, Dieynaba S. N’Diaye

**Affiliations:** 1 Action Contre la Faim, Paris, France; 2 Université de Montpellier, Montpellier, France; 3 Action Contre la Faim, Toronto, Canada; 4 Action Contre la Faim, London, United Kingdom; 5 Action Against Hunger, New York, NY, United States of America; 6 Université FHB Cocody-Abidjan, Abidjan, Côte d’Ivoire; International Maize and Wheat Improvement Centre: Centro Internacional de Mejoramiento de Maiz y Trigo, MEXICO

## Abstract

**Background:**

Acute malnutrition is one of the main causes of morbidity and mortality among children under 5 years worldwide, and Action Contre la Faim (ACF) aims to address its causes and consequences. To better tailor humanitarian programs, ACF conducts standardized contextual studies called Link NCAs (Nutrition Causal Analysis), to identify factors associated with severe acute malnutrition (SAM) and moderate acute malnutrition (MAM). Data from three Link NCAs performed in 2018 and 2019 in Haiti, Burkina Faso and Madagascar were used to explore the prevalence of malnutrition by different indicators and associated risk factors among children aged 6–59 months.

**Methods:**

Cross-sectional data, collected via household surveys applying two-stage cluster sampling, were pooled to build a sample of 1,356 children. Recommended anthropometric thresholds were used to define SAM (Weight-for-Height Z-score (WHZ) <-3 or Mid-upper Arm Circumference (MUAC) <115 mm and/or presence oedema), MAM (-3≤WHZ<-2 or 115≤MUAC<125 mm) and global acute malnutrition GAM (SAM or MAM) among children. Multivariate analyses for each anthropometric indicator were performed using logistic mixed models and adjusting for potential confounders.

**Results:**

The prevalence of acute malnutrition was the highest in Madagascar. The risk of having GAM and MAM varied across countries, while the risk of having SAM varied across clusters. Being male, suffering from diarrhea, and having unwashed face and hands, were significantly associated with GAM by WHZ with adjusted odds ratio of 1.9 [95%Confidence interval (CI):1.1–3.2], 1.7 (95%CI: 1.0–3.1) and 1.9 (95%CI: 1.0–3.6) respectively. These factors were also associated with MAM by WHZ. None of the studied factors was significantly associated with SAM, which could be due to a small sample size.

**Conclusion:**

These results obtained from a large sample contribute to the evidence of the factors associated with undernutrition in children aged 6–59 months. Further research with larger sample sizes is needed to confirm these results.

## Introduction

Acute malnutrition is one of the main causes of morbidity and mortality among children under 5 years of age worldwide. In 2018, over 49 million children under 5 year of age were wasted and nearly 17 million were severely wasted [1]. It is estimated that more than one in three deaths among children under five are attributable to undernutrition [2]. Children under the age of 5 years in low-income countries are generally the most affected. Africa accounts for more than 14 million children under 5 years with wasting [1].

As illustrated in the UNICEF conceptual framework of the determinants of child undernutrition, the causes of malnutrition are multiple and hierarchically interrelated [3]. The two immediate causes of malnutrition are inadequate dietary intake and disease, and their interaction leads to a vicious cycle whereby malnourished children are less resistant to illness, experience loss of appetite, which increases malnourishment. Dietary intake and illness are driven by numerous underlying factors [4]. Famines caused by wars and natural disasters, low socioeconomic status, household food insecurity, lack of access to water and sanitation facilities, poor quality of health services, inadequate maternal and child care and inadequate breastfeeding practices, are known contributors to malnutrition [5–7]. These factors are further influenced by the environmental, economy, politics, governance and sociocultural conditions [8].

In Burkina Faso, 7.6% of children under 5 years of age had acute malnutrition with 1.4% presenting severe acute malnutrition (SAM) according to the 2016 National Nutrition Survey [9]. Climate change, high levels of poverty coupled with rapid population growth are believed to be the major causes of food insecurity in the country. Furthermore, lack of investments in the rural areas of the country has led to insufficient food production and limited access to safe drinking water and sanitation. Inadequate family practices (hygiene and sanitation, birth spacing, etc.) and insufficient social protection for the most vulnerable are among the main factors contributing to malnutrition in Burkina Faso [10].

In Madagascar, chronic malnutrition is a major public health and development problem. According to the UNICEF, the country is the 5th most affected country in the world with 47% of children under 5 years of age (approximately 2 million children) stunted, mostly in rural areas [11]. Global acute malnutrition (GAM) affects more than 6% and SAM affects 1% of children under 5 years of age [12]. The political crisis of 2009 and recurrent droughts over the past 10 years have eroded the socio-economic capacity of the population and some households have struggled to rebuild their capital and resilience [13]. Water sources have been depleted, agricultural productivity remains low and the reduction of household purchasing power prevents adequate dietary consumption.

In Haiti, 23% of children under 5 years of age suffer from chronic malnutrition and 6% suffer from acute malnutrition. Despite efforts in chronic malnutrition reduction in recent decades, the 2019 national SMART (Standardized Monitoring and Assessment of Relief and Transitions) survey reported a slight increase in the prevalence of GAM (6%) compared to 2016–2017 estimates (4%) [14]. The combined effects of the El Niño phenomenon on agricultural production in 2014 and 2015 and the depreciation of the Haitian Gourde (the national currency) on the prices of imported food products have contributed to an increase in the number of people suffering from severe food insecurity from 600,000 in 2013 to more than 1.6 million at the end of 2015. In October 2016, Hurricane Matthew devastated some parts of Southwestern Haiti. As of 2020, an estimated 4.1 million people in the country were in acute need of food assistance [15].

Action Contre la Faim (ACF), is an international humanitarian organization aiming to eradicate child malnutrition. To inform its programs, a methodology called the Link NCA (Nutrition Causal Analysis) [16] was developed to investigate the risk factors of malnutrition in areas where ACF and other humanitarian actors operate. The Link NCA was developed by ACF in partnership with academic researchers from Tufts University and the French Research Institute for Development [16]. It is used as a contextual analysis for improving the relevance and effectiveness of multi-sectoral nutrition security programming in a given context. Initially, the Link NCA approach was designed as a tool with a strong qualitative component to identify potential associations between malnutrition and its risk factors based on the consultation of the affected communities. Recently, the SMART survey methodology [17] has been increasingly implemented alongside the community consultation component, to produce more robust quantitative estimates of the prevalence of malnutrition and to strengthen the power of the analysis of risk factors of malnutrition. By combining both qualitative and quantitative data collection and analysis, the Link NCA aims to study the prevalence and severity of malnutrition, the prevalence of known risk factors and demonstrate their association with under-nutrition among the studied population [16]. Overall, it aims to propose causal pathways of under-nutrition that are plausible and could be addressed in contexts where ACF and partners intervene.

Three Link NCAs with a standardized quantitative data collection were performed in Burkina Faso, Madagascar and Haiti in 2018 and 2019. This provided a large pooled sample and offered the opportunity to explore risk factors not just specific to one context.

Therefore, this study aims to identify common risk factors of malnutrition across three different contexts, and studying the variability in the associations according to the severity of malnutrition identified by various anthropometric indicators.

## Materials and methods

Three Link NCA quantitative surveys applying the SMART methodology were implemented between 2018 and 2019. In Burkina Faso, data were collected from September 17 to October 9, 2018, in Madagascar from March 20 to April 8, 2019, and in Haiti from May 1 to 17, 2019. Quantitative data from the three surveys were pooled to explore factors associated with malnutrition.

### Study areas and populations

The study was conducted in three different context: 1) The entire province of Kénédougou in Burkina Faso, 2) The District of Amboasary Sud in the Anosy region of Madagascar, 3) The communes of Anse d’Hainault, Dame Marie and Les Irois In Haiti, in the Grande Anse region Haiti. A detailed description of these contexts is presented in S1 Table.

### Sampling

A two-stage cluster sampling method was applied for all three surveys. For each quantitative survey, sampling and data collection were conducted following the SMART Methodology standard procedures [17]. The first sampling stage was the selection of clusters from a list of the smallest geographic units with population estimates using the ENA for SMART software (9th July, 2015 version) in accordance with the probability proportional to size principle. In Burkina Faso, the smallest geographic units were enumeration areas defined by *Institut national de la statistique et de la démographie (INSD)*, each with an average size of 1,200 people in rural areas and 1,000 people in urban areas. For Madagascar, the smallest geographic units considered in the sampling were *Fokontany* (traditional Malagasy villages) which included either settlements, villages, areas or neighbourhoods. For Haiti, the smallest geographic units were units of enumeration defined by *Ministère de la Santé Publique et de la Population*. For the three surveys, the population size for the enumeration units was updated in cooperation with local authorities before sampling by the software. Then, a segmentation method was used in enumeration units which included more than 150 households or which were geographically too large. The geographical units corresponded to an average of 700 individuals. A complete list of the smallest geographic units was compiled, any inaccessible areas due to conflict or geography were removed, and the resulting list constituted the sampling frame for each quantitative survey.

At the second stage of sampling, at cluster level, households were randomly selected from a complete list of households using simple random sampling in Madagascar, and systematic random sampling in Burkina Faso and Haiti. In each selected household, all children under five years of age were eligible to participate in the survey. However, this analysis was conducted only among children 6–59 months of age, and Mid-upper Arm Circumference (MUAC) measurements were not performed for children under six months of age.

S2 Table of shows the parameters considered for the calculation for the sample size.

### Data collection

The collection of quantitative data in the three countries was done using questionnaires which were designed, consolidated, and validated by the survey manager and operational actors from various sectors of interest with input from local stakeholders [including health, nutrition, food security and livelihoods, water, sanitation and hygiene (WASH), and education]. A sample Link NCA questionnaire can be found in the S1 Appendix. The questionnaires were translated into the most prominent local language in Madagascar (Malagasy) and Haiti (Haitian). The questionnaires were administered electronically using the KoboCollect application [18] on tablets. Data were collected and entered into tablets in real time and uploaded to a remote server and downloaded daily by the survey manager to monitor data quality.

Across all surveys, data on retrospective mortality, food security, livelihoods, and WASH were collected at household level. Among women 15–49 years of age, health data were collected. Among children 6–59 months of age, data on health, infant and young child feeding practices, and anthropometric measurements (height, weight, MUAC, presence of oedema) were collected.

### Acute malnutrition assessment

Anthropometric measurements were taken from all eligible children in order to assess acute malnutrition by three main indicators: Weight-for-Height Z-score (WHZ), MUAC, and bilateral pitting oedema. The outcomes of interest were acute malnutrition per WHZ, acute malnutrition per MUAC, and acute malnutrition per combined WHZ and/or MUAC (Table 1).

**Table 1:** Acute malnutrition criteria and thresholds.

	GAM	MAM	SAM
WHZ	Z-score <-2 and/or oedema	-3 ≤ Z-score <-2	Z-score < -3 and/or oedema
MUAC	MUAC <125 mm and/or oedema	115mm ≤ MUAC <125 mm	MUAC <115 mm and/or oedema
Combined variables	Z-score <-2 and/or MUAC <125 mm and/or oedema	-3 ≤ Z-score <-2 and/or 115mm ≤ MUAC <125 mm	Z-score < -3 and/or MUAC <115 mm and/or oedema

Abbreviations: GAM: Global Acute Malnutrition; MAM: Moderate Acute Malnutrition; MUAC: Mid-upper Arm Circumference; SAM: Severe Acute Malnutrition; WHZ: Weight for Height Z-score

Recommended thresholds for anthropometric measurements were used to define SAM (WHZ <-3 or MUAC <115 mm and/or presence oedema), moderate acute malnutrition (MAM) (-3≤WHZ<-2 or 115≤MUAC<125 mm without oedema) and GAM (SAM or MAM) among children [19–21] (Table 1).

For the combined variables, combined GAM (cGAM) is defined as all cases of GAM by WHZ and/or MUAC and/or oedema [19, 20]. Combined MAM (cMAM) is defined as all cases of MAM by WHZ and/or MUAC without oedema, and combined SAM (cSAM) as all cases of included SAM by WHZ and/or MUAC and/or oedema [21].

### Explanatory variables

Risk factors that were likely to be associated with acute malnutrition based on either published or grey literature, were validated by technical experts during an initial workshop were collected as part of the quantitative survey. Only comparable variables collected across all three surveys were retained for this analysis and they were grouped into three categories as per the UNICEF conceptual framework of the determinants of child undernutrition:

1. Child characteristics: sex of the child [22], vitamin A supplementation per six-month recall (yes / no) [23], deworming treatment per six-month recall (yes / no) [24], diarrhea per two-week recall (yes / no) [22, 23], fever per two-week recall (yes / no) [25], hand and face cleanliness of child (observation) (yes / no) [26], cleanliness of child’s clothes (observation) (yes / no) [26],
2. Household characteristic: sex of the head of household (male / female) [27], large family (number of family members higher than the national average) (yes / no) [28], lack of sufficient food for the household per 12-month recall (yes / no) [22, 25],
3. Environmental characteristic: place of birth of the youngest child (home / outside of home) [29], number of antenatal visits during the last pregnancy (<4 / ≥4) [28, 30] and protected main drinking water source (yes / no) [25].

Assessing the main drinking water source was based on a comprehensive list of possible water sources adapted to each country context. Sources were classified and analysed as protected *versus* non-protected. In order to harmonize the variable for all countries, this reclassification of the variable as a protected and unprotected main drinking water source was done with the help of a WASH specialist (see S3 Table). The variable "place of birth of the last child" was also based on a list of responses adapted to each country. Responses were reclassified as birth at home or birth outside the home. All other variables were standardized global indicators. Potential risk factor variables that could not be harmonized for statistical comparison were not included in this analysis.

### Statistical analysis

Anthropometric variables by WHZ (GAM, MAM and SAM), MUAC (GAM, MAM and SAM) and combined categories (cGAM, cMAM, cSAM) were structured as binary outcomes (positive or negative) for the analysis. The prevalence of each anthropometric variable was calculated for each survey area. A univariate descriptive statistical analysis of anthropometric variables and risk factor variables for each of the three studies was conducted.

Several logistic regression models, classical models (with fixed effect only) and random effect models were used for each anthropometric variable. As a level of heterogeneity was observed between clusters and by country, these last two variables were introduced into the models as a random effect [31]. Despite a low number of modalities, as the random effects represent a higher level variable under which data points are grouped, the country can therefore be considered a random effect.

Four types of models were tested: a model without random effect, a model with *cluster* as random effect, a model with *country* as random effect, and a model with *cluster* and *country* as random effect. The variance components of each model were tested with the likelihood ratio test. The models were tested in pairs and the best model was the one with *cluster* and *country* as random effect. Furthermore, the result of the Akaike information criterion (AIC) also identify the model with cluster and country in random effect as the best suited model.

First, univariate models were carried out on the pooled data to study the association between the anthropometric variable and the potential risk factors listed previously. Second, multivariate analyses for each anthropometric category were performed. The independent variables introduced in the multivariate models are the variables with a p-value < 0.20 at least once in the univariate models of the three anthropometric categories. For example, for the three multivariate models for the WHZ category (GAM, MAM and SAM), the explicative variables introduced in the models were all variables with a p-value < 0.20. For models with SAM by WHZ or SAM by WHZ & MUAC combined as outcome, the best models are those with *cluster* as random effect. For models with SAM by MUAC as outcome, the best models are the models without random effects. For the other models, it remains with cluster and country in random effects (further detail can be found in the S5 Table). For all analyses, associations were considered statistically significant when the p-value was < 0.05.

A calculation of the percentage of missing values was made. The number of missing data was reduced, and the fact of having missing data was considered to be random. For that reason, the complete case analysis was used to treat the missing data [32]. All analyses were carried out with the R Studio software version 3.6.1.

### Ethical considerations

The Link NCAs were each reviewed and approved by an institutional review committee prior to the beginning of each study. In Haiti, permission to conduct the survey was obtained from the Nutrition Unit of the Ministry of Public Health and Population (Unité de Coordination du Programme National d’Alimentation et de Nutrition (UCPNANu)). In Madagascar, permission was obtained from the regional office of the *Office National de Nutrition (ONN)* and the Regional Department of Health. In Burkina Faso, permission was obtained from the *Direction Nationale de la Nutrition*. A new approval was not requested to conduct these analyses, which were secondary in nature. Further, a new authorization was not necessary because the data are anonymous and did not include any information that would enable to identify participants [33].

After describing the survey, its objectives and duration in the local language, informed consent was obtained verbally (rather than in writing for ethical reasons that apply in the case of illiterate participants) from the head of household and entered in the tablets before data collection began. Heads of households and mothers of children under five years of age provided oral consent to participate in the survey as well as permission for the participation of all children under five years of age in the household. All the answers were recorded in a way that ensured and maintained anonymity and confidentiality. Participants were allowed to ask questions before giving their consent or refusal to participate.

## Results

### Socio-demographic characteristics

The Link NCA quantitative surveys collected data on 395 children in Haiti, 481 children in Madagascar and 441 children in Burkina Faso between the ages of 6 and 59 months. All children were retained for analysis. Table 2 describes the characteristics of the study population. In all three survey areas, females slightly outnumbered males in the sampled children (52% in Burkina Faso, 53% in Haiti and 53% in Madagascar) and the average age among children surveyed was 32 months (standard deviation (SD) = 15) in pooled sample (33 months (SD = 15) in Burkina Faso, 32 months (SD = 15) in Haïti and 31 (SD = 15) in Madagascar). In Haiti, 41.8% of households surveyed were female-headed, compared to 37.6% in Madagascar and 12.3% in Burkina Faso. Lack of food to meet the family’s needs within the last 12 months was reported most frequently in Haiti (93.6% of households) and in Madagascar (93.3%). In Burkina Faso, only 12.8% of households reported not having enough food to meet family needs in the last 12 months. Most children surveyed had received vitamin A and Albendazole supplementation in the last 6 months. Only in Haiti were the majority of children classified as having a good hygiene (observation of children with clean face, hands and clothes).

**Table 2:** Characteristics of the study population by country and overall.

			Total (N = 1,317)	Burkina Faso (N = 441)	Madagascar (N = 481)	Haiti (N = 395)
			N (%)	N (%)	N (%)	N (%)
Characteristics of the child	Sex of the child					
		*Female*	694 (53)	227 (52)	256 (53)	211 (53)
		*Male*	623 (47)	214 (49)	225 (47)	184 (47)
	Age of the child					
		*6–11 months*	119 (9)	27 (6)	51 (11)	41 (10)
		*12–23 months*	322 (24)	105 (24)	124 (26)	93 (24)
		*24–35 months*	299 (23)	111 (25)	101 (21)	87 (22)
		*≥ 36 months*	577 (44)	198 (45)	205 (43)	174 (44)
	Vitamine A^1^					
		*Yes*	1007 (80)	361 (82)	380 (82)	266 (73)
		*No*	259 (20)	80 (18)	81 (18)	98 (27)
	Deworming^2^					
		*Yes*	818 (72)	320 (79)	309 (76)	189 (59)
		*No*	313 (28)	87 (21)	96 (24)	130 (41)
	Diarrhea per two-week recall					
		*No*	921 (71)	370 (84)	298 (63)	253 (65)
		*Yes*	380 (29)	71 (16)	175 (37)	134 (35)
	Fever per two-week recall					
		*No*	797 (61)	330 (75)	237 (50)	230 (60)
		*Yes*	503 (39)	111 (25)	236 (50)	156 (40)
	Clean face and hands^3^					
		*No*	756 (58)	269 (62)	324 (69)	163 (42)
		*Yes*	539 (42)	167 (38)	145 (31)	227 (58)
	Clean clothes					
		*No*	838 (65)	290 (66)	372 (79)	176 (46)
		*Yes*	447 (35)	146 (34)	96 (21)	205 (54)
Characteristics of the household	Sex of the head of household					
		*Male*	908 (69)	378 (88)	300 (62)	230 (58)
		*Female*	399 (31)	53 (12)	181 (38)	165 (42)
	Large family (> country average^4^)					
		*No*	693 (53)	251 (57)	260 (54)	182 (46)
		*Yes*	624 (47)	190 (43)	221 (46)	213 (54)
	Inadequate household food provisioning per 12-month recall					
		*No*	432 (34)	376 (87)	32 (7)	24 (6)
		*Yes*	851 (66)	55 (13)	443 (93)	353 (94)
Other environmental characteristics	Water source					
		*Protected*	365 (28)	124 (29)	127 (27)	114 (30)
		*No protected*	925 (72)	306 (71)	348 (73)	271 (70)
	Birth location					
		*Home*	362 (32)	43 (10)	98 (25)	221 (74)
		*Out of home*	760 (68)	388 (90)	295 (75)	77 (26)
	Prenatal consultation < 4					
		*No*	719 (65)	242 (56)	297 (78)	180 (60)
		*Yes*	390 (35)	189 (44)	83 (22)	118 (40)

^1^ Receiving vitamin A supplementation in the last 6 months.

^2^ Receiving Albendazole in the last 6 months.

^3^ Observed by enumerators.

^4^ country average: for each country, it is the mean of the household members included in the study.

### Prevalence of acute malnutrition

**Fig 1:**
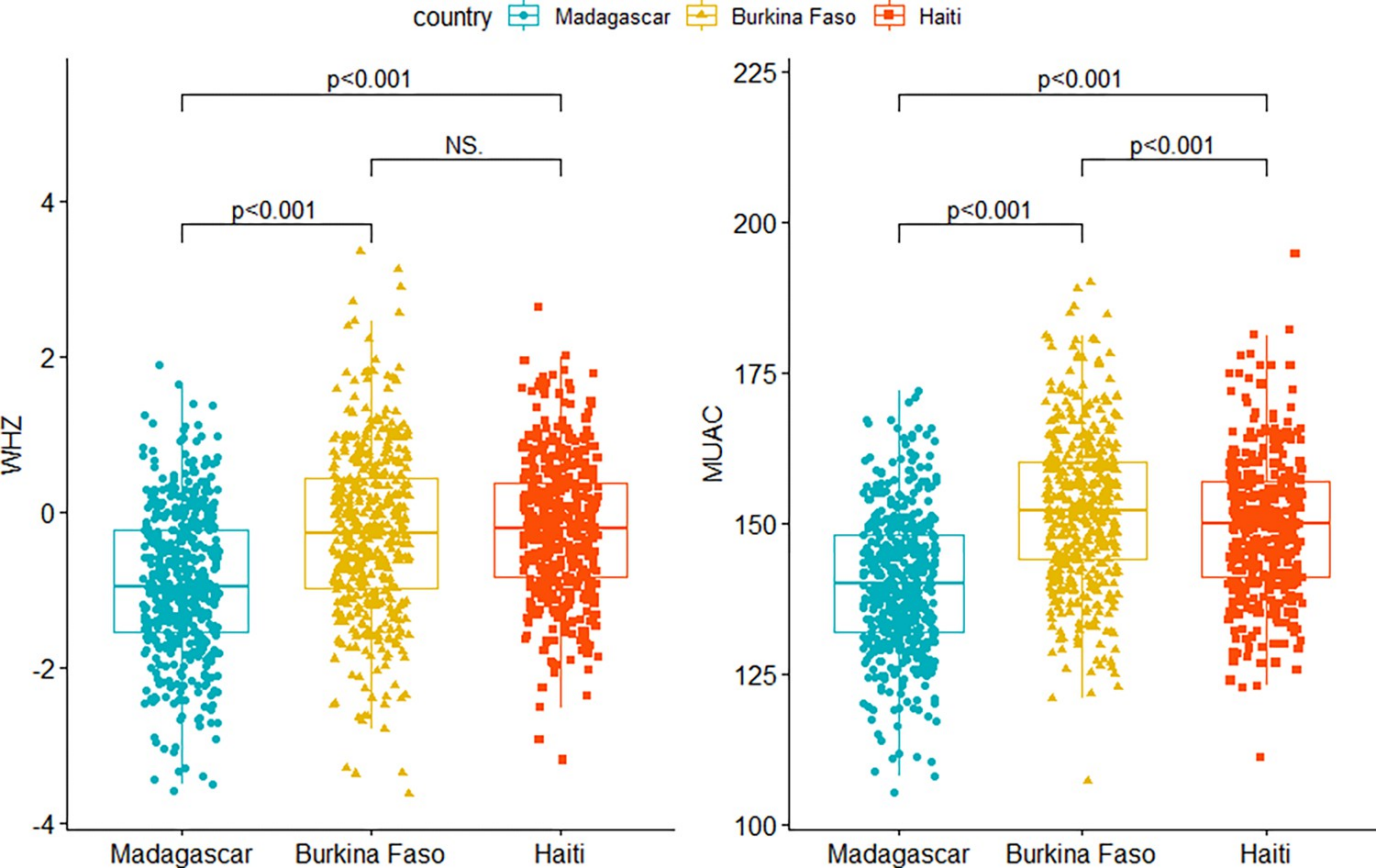
Distribution and comparisons of anthropometric measures WHZ and MUAC in the three countries.

According to the WHO thresholds, among the 1,317 children with anthropometric measurement across the three countries, 87 (6.6%) children had GAM by WHZ including 16 (1.2%) with SAM by WHZ. Furthermore, 43 (3.2%) children had GAM by MUAC and 11 (0.8%) had SAM by MUAC. The prevalence of acute malnutrition by all anthropometric indicators was found to be higher in Madagascar than in the other two countries (Table 3). MUAC increased with age in the three countries while it was not the case for WHZ except for Burkina Faso. Graphics illustrating the distribution of MUAC and WHZ according to age can be seen in S1 and S2 Figs. A significant difference was found between the anthropometric measures (WHZ and MUAC) in the three countries except, for WHZ which was similar in Burkina Faso and Haiti (Fig 1). In all three survey areas, the prevalence of acute malnutrition was higher for combined indicators (8.0%) and per WHZ (6.6%) than per MUAC (3.3%), although differences in prevalence were not significant. Two cases of oedema were identified in Haiti while none was reported in Madagascar or Burkina Faso. If only MUAC had been used to detect SAM, 63% of children with SAM by WHZ would have been missed in the overall sample. Alternatively, if only WHZ had been used to detect SAM, 45% of children with SAM by MUAC would have been missed.

**Table 3:** Prevalence of acute malnutrition in the study population.

		Total (N = 1,317)	Burkina Faso (N = 441)	Madagascar (N = 481)	Haiti (N = 395)
		N (Prevalence)	N (Prevalence)	N (Prevalence)	N (Prevalence)
WHZ					
	GAM	87 (6.6)	21 (4.8)	57 (11.9)	9 (2.3)
	MAM	71 (5.4)	17 (3.9)	48 (10.0)	6 (1.5)
	SAM	16 (1.2)	4 (0.9)	9 (1.9)	3 (0.8)
MUAC					
	GAM	43 (3.3)	4 (0.9)	34 (7.1)	5 (1.3)
	MAM	32 (2.4)	3 (0.7)	26 (5.4)	3 (0.8)
	SAM	11 (0.8)	1 (0.2)	8 (1.7)	2 (0.5)
WHZ & MUAC					
	cGAM	105 (8.0)	22 (5.0)	71 (14.8)	12 (3.0)
	cMAM	89 (6.8)	18 (4.1)	62 (12.9)	9 (2.3)
	cSAM	21 (1.6)	4 (0.9)	14 (2.9)	3 (0.8)

WHZ: Weight for Height Z-score

MUAC: Mid-upper Arm Circumference.

GAM: Global Acute Malnutrition

MAM: Moderate Acute Malnutrition

SAM: Severe Acute Malnutrition

### Factors associated with acute malnutrition (univariate analysis)

The univariate model results are presented in S4 Table. Sex, the presence of fever and a large family size were found to be significantly associated with GAM by WHZ. Surprisingly, large family size was the only variable that was significantly associated with the GAM by all three indicators (GAM by WHZ, GAM by MUAC and cGAM) but in negative way. A child from a larger family than the national average was less at risk of having a GAM. The presence of diarrhea was found to be significantly associated with MAM by all three indicators (MAM by WHZ, by MUAC and by cMAM). No significant association was found between the different variables and SAM by WHZ. In contrast, sex of the child and vitamin A supplementation were significantly associated with SAM by MUAC. Males had a higher risk of GAM by WHZ and MAM by WHZ compared to females. Females on the other hand were found to be more at risk of SAM by MUAC than males.

### Factors associated with acute malnutrition (multivariate analysis)

The results of the multivariate analyses can be found in Table 4. No significant association was found between potential risk factors and SAM according to the 3 indicators (WHZ, MUAC and combined WHZ and MUAC). Being male, suffering from diarrhea, and having unwashed face and hands significantly increased the probability of GAM by WHZ. Similar associations were found for MAM by WHZ. In contrast, using an unprotected water source was negatively associated with GAM by WHZ. In addition, suffering from fever increased the risk of MAM by WHZ. For MUAC, only diarrhea was found to be significantly associated with GAM. Children with diarrhea per two-week recall were 10.4 (CI 95%: 2.0–54.5) times more likely to have GAM by MUAC. Regarding the combined indicators, children with diarrhea per two-week recall were 2.1 times (CI 95%: 1.2–3.6) more likely to have cGAM, while an unprotected water source significantly decreased the risk of cGAM by 60%. Similar associations were found for cMAM. In addition, male sex (with an OR of 1.8, CI 95%: 1.0–3.2) and having face and hands not washed increasing the risk (OR = 2.0, CI 95%: 1.0–3.9) of having cMAM.

**Table 4:** Multivariate models between anthropometric categories and risk factors for malnutrition.

		WHZ (N = 1,060)^1^						MUAC (N = 945)^1^						combined WHZ and MUAC (N = 938)^1^					
	
		GAM		MAM		SAMⴃ		GAM		MAM		SAMⴉ		cGAM		cMAM		cSAM ⴃ	
		OR	CI 95%	OR	CI 95%	OR	CI 95%	OR	CI 95%	OR	CI 95%	OR	CI 95%	OR	CI 95%	OR	CI 95%	OR	CI 95%
Sex of the child -	*Female (ref)*	1.0		1.0		1.0		1.0		1.0		1.0		1.0		1.0		1.0	
	*Male*	**1.9**	**(1.1–3.2)** *	**2.2**	**(1.2–3.8)** **	0.9	(0.3–3.3)	0.5	(0.1–1.9)	0.9	(0.3–2.7)	**-**	-	1.7	(1.0–2.8)	**1.8**	**(1.0–3.2)** *	1.0	(0.3–3.6)
Vitamin A -	*Yes (ref)*	1.0		1.0		1.0		1.0		1.0		1.0		1.0		1.0		1.0	
	*No*	1.2	(0.6–2.3)	0.8	(0.4–1.7)	3.5	(0.9–14.5)*	0.8	(0.1–7.2)	0.5	(0.1–3.6)	11.6	(0.7–503.5)	1.2	(0.5–3.0)	0.9	(0.3–2.3)	9.5	(0.7–120.8)*
Deworming -	*Yes (ref)*							1.0		1.0		1.0		1.0		1.0		1.0	
	*No*							5.2	(0.6–44.2)	2.9	(0.6–13.8)	1.5	(0.1–58.3)	1.3	(0.6–3.0)	1.6	(0.7–3.7)	0.4	(0.0–4.8)
Diarrhea per two-week recall	*No (ref)*	1.0		1.0		1.0		1.0		1.0		1.0		1.0		1.0		1.0	
	*Yes*	**1.7**	**(1.0–3.1)** *	**1.8**	**(1.0–3.2)** *	1.4	(0.3–6.1)	**10.4**	**(2.0–54.5)** **	3.4	(0.9–12.3)	3.1	(0.3–28.2)	**2.1**	**(1.2–3.6)** *	**2.0**	**(1.1–3.6)** *	1.4	(0.3–6.3)
Fever per two-week recall	*No (ref)*	1,0		1,0		1,0								1.0		1.0		1.0	
	*Yes*	1.6	(0.9–2.9)	**2.2**	**(1.2–3.9)** *	0.4	(0.1–2.1)							1.2	(0.7–2.1)	1.4	(0.8–2.5)	0.8	(0.2–3.7)
Clean face and hands	*Yes (ref)*	1.0		1.0		1.0		1.0		1.0		1.0		1.0		1.0		1.0	
	*No*	**1.9**	**(1.0–3.6)** *	**2.1**	**(1.1–4.2)** *	0.8	(0.2–3.7)	8.6	(0.0–1835.5)	12.4	(0.2–753.8)	-	-	1.8	(0.9–3.4)	**2.0**	**(1.0–3.9)** *	1.2	(0.2–5.7)
Clean clothes -	*Yes (ref)*							1.0		1.0		1.0							
	*No*							1.0	(0.0–212.1)	0.5	(0.0–36.6)	-	-						
Female head of household -	*No (ref)*																		
	*Yes*																		
Large family (>country average) -	*No (ref)*	1.0		1.0		1.0		1.0		1.0		1.0		1.0		1.0		1.0	
	*Yes*	0.7	(0.4–1.2)	0.8	(0.4–1.4)	0.5	(0.1–2.0)	0.2	(0.0–1.4)	0.3	(0.1–1.3)	1.0	(0.1–9.1)	0.8	(0.4–1.3)	0.8	(0.4–1.4)	0.8	(0.2–3.3)
Inadequate household food provisioning per 12-month recall	*No (ref)*																		
	*Yes*																		
Water source -	*Protected (ref)*	1.0		1.0		1.0								1.0		1.0		1.0	
	*No protected*	**0.5**	**(0.3–0.9)** *	**0.5**	**(0.3–1.0)** *	0.6	(0.1–2.3)							**0.4**	**(0.2–0.8)** **	**0.5**	**(0.3–0.9)** *	0.5	(0.1–2.1)
Birth location -	*Out of home (ref)*																		
	*Home*																		
pre-natal consultation < 4 -	*No (ref)*	1.0		1.0		1.0		1.0		1.0		1.0		1.0		1.0		1.0	
	*Yes*	1.3	(0.7–2.3)	1.3	(0.7–2.5)	1.0	(0.2–3.9)	0.3	(0.1–1.5)	0.4	(0.1–2.1)	1.7	(0.2–14.7)	1.3	(0.7–2.2)	1.3	(0.7–2.5)	1.0	(0.2–4.1)

***p-value < 0.001

**p-value < 0.01

*p-value < 0.05

: Variable not included in the model

Ref: the reference class for a qualitative variable

OR: Odds ratio

CI 95%: confidence interval at 5% alpha risk

ⴉ: Model without random effect

ⴃ: Model with only cluster as random effect

WHZ: Weight for Height Z-score

MUAC: Mid-upper Arm Circumference

MAM: Moderate Acute Malnutrition (for WHZ: -3≤WHZ<-2; For MUAC: 115≤MUAC<125 mm)

SAM: Severe Acute Malnutrition (for WHZ: WHZ <-3 and/or presence oedema; For MUAC: MUAC <115 mm and/or presence oedema)

GAM: Global Acute Malnutrition (SAM or MAM)

cGAM: combined Global Acute Malnutrition

cMAM: combined Moderate Acute Malnutrition

cSAM: combined Severe Acute Malnutrition.

^1^ Unlike in Tables 2 and 3, N in Table 4 corresponds to the number of subjects who had complete data on the dependent variable and risk factors

Analyses using the two different models (univariate and multivariate) demonstrated that the risk of having GAM (by WHZ, by MUAC or cGAM) or MAM (WHZ, MUAC, or cMAM) varied between countries (see S4 Table). This means that the local context itself has an effect on the probability for a child to be affected by GAM or MAM. However, the risk of having SAM by WHZ and cSAM varied only between clusters. No cluster or country effect was found for SAM by MUAC.

## Discussion

This study examined cross-sectional data collected from Burkina Faso, Madagascar and Haiti on a pooled sample of 1,317 children aged 6–59 months allowing to study the WHZ and MUAC specific prevalence of and characteristics associated with acute malnutrition across the three contexts; however, the number of SAM cases was low. Results indicated a non-significant difference in the prevalence of GAM, MAM and SAM between WHZ and MUAC based indicators whereby WHZ based classification resulted in higher prevalence of malnutrition compared to MUAC. Furthermore, different risk factors were associated with acute malnutrition by WHZ and by MUAC.

Previous studies have demonstrated that WHZ and MUAC indicators do not necessarily capture the same populations [34–36], that prevalence is generally higher by WHZ than by MUAC, especially in crisis contexts [34] and that the relationship between the two indicators varies. The specificity of MUAC and WHZ to detect children at risk of death have been reported to be high for both indicators (>95%), however, correlations between MUAC and WHZ have been found to be weak [37]. According to Berkley *et al*. (2005), clinical features of malnutrition or malaria parasitemia were significantly more common among children with MUAC ≤ 115 mm than among those with WHZ ≤ −3 [38]. However, a study from Dailey-Chwalibóg *et al*. (2021) on children with SAM aged 6 to 59 months in Bangladesh, Burkina Faso, and Liberia showed that children with WHZ < - 3 and MUAC ≥ 115 mm had equally or more severe deficits in nutritional status, hydration, and iron balance than children with WHZ ≥ -3 and MUAC < 115 mm, and presented lower levels of leptin, a marker of mortality risk among children with SAM [39].

The results from this study demonstrated that the prevalence of malnutrition was highest in Madagascar, followed by Burkina Faso and finally Haiti. The prevalences of GAM by WHZ in each country was slightly lower but close those of previous studies in the same areas: 12.9% in Amboasary in Madagascar in 2018 [40] *versus* 11.9% in the current study, 7.8% in 2017 in Kénédougou in Burkina Faso [9] *versus* 4.8% in the current study, and 5% in 2018 in the Grande Anse region in Haiti [14] *versus* 2.3% in the current study. Although all three areas are parts of low-income countries, they do not have the same socio-demographic, economic and environmental specificities. Child characteristics were found to be worse in Madagascar than in the other two countries: low vitamin A supplementation coverage, lower personal hygiene (face, hands and clothes) scores and higher prevalence of illness such as fever and diarrhea. This is coherent with studies documenting that wasting is often associated with a deteriorated health status [22]. Diseases like diarrhea have been shown to be associated with malnutrition [22].

The factors found to be significantly associated with GAM by WHZ in all three pooled countries were being male, suffering from diarrhea, having unwashed face and hands, and using a protected water source. Only diarrhea was found to be significantly associated with GAM by MUAC. For GAM from combined indicators, risk factors were diarrhea and a protected water source. By distinguishing MAM and SAM, we found no significant association between potential risk factors and SAM according to the 3 indicators and MAM by MUAC. On the other hand, being male, suffering from diarrhea, having unwashed face and hands and using a protected water source were significantly associated with MAM by WHZ and MAM by combined indicators. Furthermore, suffering from fever was associated with MAM by WHZ.

These findings on the significant predictors of malnutrition are aligned with results from Link NCAs’ qualitative surveys on the communities’ perceptions of risk factors and underlying mechanisms of malnutrition. In Madagascar and Haiti [13, 41], participants of the community consultation mentioned their own poor nutritional status as a reason for their reduced ability to breastfeed their children, especially boys, who are given complementary food at a very early age and are therefore exposed to infections and possibly to the risk of undernutrition. Other important risk factors identified by participants in the three countries were poor access to water and poor personal hygiene and sanitation practices, which in turn often lead to recurrent episodes of diarrhea [13, 41] (Burkina Faso data are not published). This alignment of the results from the quantitative and qualitative surveys speak to the benefit of investigating the community’s own perceptions on acute malnutrition in order to enhance program design in humanitarian contexts.

Moreover, the significant association between sex of the child (male) and GAM/MAM by WHZ demonstrated by this analysis is consistent with several studies that have shown that males have a higher risk than females to be affected by GAM or/and MAM by WHZ [42]. A systematic review conducted in sub-Saharan countries including 49 studies selected based on their methodological robustness reported that 35% of the studies showed an association between the male sex and GAM and/or MAM by WHZ [22]. This might be explained by different care practices. Indeed, several Link NCAs have shown that in the Sahel, males tend to be more quickly weaned from their mothers (physically, emotionally, and from milk). In Madagascar, a survey showed that women had perceived an insufficiency of breast milk when breastfeeding a male child [13] although no significant association was found between sex of the child and GAM/MAM by MUAC. The low prevalence of GAM and MAM by MUAC may explain the absence of a significant association between the sex of the child and GAM/MAM by MUAC. Prevalence of GAM by MUAC was less than 2% in Burkina Faso and Haiti. Only Madagascar has a prevalence above 5% for MAM by MUAC.

A significant association was found between the sex of the child and SAM by MUAC with a higher risk of malnutrition in females than males in the univariate model only. However, our sample contained a small number of SAM cases, therefore results should be interpreted cautiously.

Results of this study suggested that suffering from diarrhea (in the 14 past days before the survey) was significantly associated with GAM and MAM for the three indicators. This is consistent with the literature, where the association between recent episode of diarrhea and malnutrition is found in several studies [22, 43, 44]. In the systematic review by Akombi *et al*. (2017), 80% of the studies showed an association between a recent episode of diarrhea and malnutrition [22]. Diarrhea is assumed to be one of the major causes of malnutrition [45] with a bidirectional interaction between the two conditions. Indeed, diarrhea can be considered both the cause and consequence of malnutrition, creating a cyclical relationship between diarrhea and malnutrition [46]. Lack of personal hygiene has also been shown in the literature to be associated to malnutrition [47–49]. It is believed that hygiene can cause malnutrition through diarrhea and through subclinical conditions like environmental enteropathy [50, 51]. This is in line with the findings that unwashed face and hands was significantly associated with GAM and MAM by WHZ and by combined indicators.

Unexpectedly, a significant negative association was found between water source and GAM/MAM by WHZ and by combined indicators indicating that unprotected source water decreases the risk of GAM and MAM by WHZ and by combined indicators. This protective effect opposes several studies that have demonstrated that an unprotected water source increases the risk of malnutrition [52, 53]. According to the WHO, unprotected water source is included among the risk factor reflective of an unhygienic environment with undernutrition [54]. Our counterintuitive finding may be the result of confounding whereby a protective factor not included in our study is correlated with water source and this way gives the false impression that a protected water source is associated with higher GAM/MAM. The water source variable was developed differently in each country and was reclassified as a protected and unprotected source (S3 Table). This might explain these results. However, other hypotheses may also be considered such as the possibility that households treat water from unprotected sources in contrast to sources that they consider protected. The result may also have been influenced by limitations in the enumerators’ ability to determine if water sources were protected or unprotected during data collection.

Most risk factors found to be associated with malnutrition in this study are related to the Water, Sanitation and Hygiene (WASH). It has been shown that exposure to inadequate WASH may result in a range of poor health outcomes including malnutrition [51]. Several studies have shown the relationship between WASH conditions and malnutrition, indeed a 2018 systematic review of 36 studies on the determinants of malnutrition in children under five in developing countries, showed that poor WASH conditions were one of the major contributors to malnutrition in children under five years of age and was associated with higher mortality [55]. It has also been shown that the absence of drinking water sources and toilet facilities were associated with malnutrition [55]. Furthermore, inadequate sanitation facilities contribute to increasing contamination of food and drinking water and children living in a house without toilets are more likely to be underweight and at risk of stunting [55]. The hypothesised pathway is that WASH may impact malnutrition by reducing infection with enteric pathogens that could then cause diarrhea [46], therefore it is plausible that poor WASH conditions increase malnutrition.

With reference to the UNICEF framework, we were able to identify a factor considered to be an immediate cause of malnutrition, namely diarrhea (disease), and another one considered to be an underlying cause of malnutrition namely unwashed face and hands (inadequate care practice). We have also identified a basic cause of malnutrition, namely sex (physiological and socio-cultural factor).

The inclusion of data obtained via two-stage cluster sampling from three countries with different socio-demographic, geographic, climatic and cultural characteristics increases the robustness and external validity of findings from this study. To our knowledge, only a few studies have evaluated the risk factors of GAM, MAM and SAM with different anthropometric indicators in the same population and none across various countries. One strength of this study was the ability to compare risk factors for malnutrition by different indicators in both univariate and multivariate models. This study demonstrated, for example, that in a univariate model, the intake of vitamin A and deworming were significantly associated with GAM by MUAC and not by WHZ.

Limitations of this study include the limitations inherent to cross-sectional studies. First, the lack of longitudinal follow-up of individuals can reduce the reliability of the results. Thus, it was not possible to identify a causal relationship between the tested factors and undernutrition. Second, potential misclassification may have occurred during the collection of retrospective information, namely regarding the occurrence of diarrhea and fever episodes that are measures of disease prone to recall biais [56]. Third, by pooling data from three different countries in three different world regions, the context-specificity of the factors contributing to acute malnutrition were lost. This also resulted in the reclassification and homogenisation of some variables like water source, which limited the evaluation method. Fourth, the period chosen for the cross-sectional data collection in each of the three countries was not necessarily representative of the rest of the year and did not allow us to examine the potential seasonality of the associations. The period (season) of data collection was different in each country and different associations could have been observed if the study had been conducted in a different season. Those differences in the data collection period might lead to a bias in these results since several studies suggest that seasonality plays an important role in risk factors for malnutrition [57]. Finally, a minimal number SAM cases were found in our sample leading to an inflation of variance in our study. This is because the number of subjects needed for the Link NCA studies in the field was calculated based on an expected prevalence of GAM (more prevalent than SAM). This could also explain the fact that we have been unable to conclude on any significant joint associations with SAM in the analysis regrouping the three countries.

This study has identified some risk factors for malnutrition generally shown in the literature. The results taking into account three samples from different regions of the world suggests that whatever the context, these variables contribute to malnutrition. However, several risk factors were not considered because the selection of potential risk factors was based on contextual and country-specific assumptions.

This study aims to support decision making in humanitarian situations where anthropometric measurements are based on the WHO thresholds that define malnutrition as a medical condition. Therefore, anthropometric variables were studied as categorized variables. Additional analyses exploring both WHZ and MUAC as continuous variables through linear mixed models did not change our main findings. Indeed, the choice of the best model (the mixed model with cluster and country as random effect) remained the same. Regarding the risk factors, diarrhea remained the only one significantly associated with a decrease in both WHZ and MUAC. For the model with MUAC as outcome, the absence of deworming was significantly associated with a decrease of MUAC—an association that was not significant when considering MUAC as a categorized variable.

This study should be considered as a pilot, and further research is needed to consolidate the results for a better orientation for field interventions against malnutrition. For future research, it will be interesting to include data from other cross-sectional surveys from different contexts and performed in the same area but at different time of the year. Furthermore, it will be important to consider the collection of a larger number of variables in each country and an expected prevalence of SAM in the calculation of sample size in order to be able to conclude on the risk factors associated with SAM. Those pooled results can be completed and improved by Link NCA data from other countries of intervention. These findings could help to better orient humanitarian interventions in the field.

The comparison of various anthropometric indicators enabled to draw attention to differences in malnutrition prevalence by different indicators (WHZ and/or MUAC) and associated risk factors. Based on these results, we can assume that improved and accessible WASH services preventing infectious diseases related to diarrhea might help reduce the risk of malnutrition regardless of the context of intervention. Additionally, interventions to promote and facilitate optimal and gender-neutral childcare practices to maintain protective hygiene levels might also help reduce the risk of malnutrition. Furthermore, comprehensive and multisectoral programming in areas with high levels of malnutrition including sensitisation to appropriate water management and hygiene practices could contribute to addressing the multicausal pathways leading to poor child health in general and undernutrition in particular. It is important to continue to collect and analyse the different indicators of malnutrition in other settings to obtain an overall and more accurate overview of the nutritional situation and the related factors, and to propose targeted and comprehensive actions.

## Conclusion

This study included a large sample of children aged 6–59 months from three different countries in order to identify the factors associated with acute malnutrition by three indicators (WHZ, MUAC and combined WHZ/MUAC). The factors associated with GAM and MAM found in this study have a high level of evidence allowing for a better representation of universal factors associated with acute malnutrition in this segment of the population (being male, suffering from diarrhea, having unwashed face and hands). This study also provided an understanding of the variation in the prevalence of GAM, MAM and SAM and the associated factors involved.

These findings deepen the knowledge of the factors associated with undernutrition in children to better guide the humanitarian interventions. This may ultimately improve the relevance of the upcoming Link NCAs and the effectiveness of multi-sector humanitarian assistance delivered by ACF and its partners.

## Supporting information

S1 Appendix Sample questionnaire administrated during Link NCA studies. (PDF)

S1 Fig Distribution of WHZ according to age. WHZ: Weight for Height Z-score; age in months.
(TIF)

S2 Fig Distribution of MUAC according to age. MUAC: Mid-upper Arm Circumference; age in months.
(TIF)

S1 Table Study areas and populations. (DOCX)

S2 Table Parameters used to calculate the sample size. (DOCX)

S3 Table Recoding of the variable main drinking water source. * Recoding as “1” corresponds to unprotected main drinking water source and “0” corresponds to protected main drinking water source.
(DOCX)

S4 Table Univariate models between anthropometric indicators and risk factors for malnutrition. ***p-value < 0.001; **p-value < 0.01; *p-value < 0.05; ●p-value < 0.20; Ref: the reference class for a qualitative variable; OR: Odds ratio; CI 95%: confidence interval at 5% alpha risk; WHZ: Weight for Height Z-score; MUAC: Mid-upper Arm Circumference; GAM: Global Acute Malnutrition; MAM: Moderate Acute Malnutrition; SAM: Severe Acute Malnutrition; cGAM: combined Global Acute Malnutrition; cMAM: combined Moderate Acute Malnutrition; cSAM: combined Severe Acute Malnutrition.
(DOCX)

S5 Table Random effects results from multivariate models. For each indicator, several random effect mixed models were tested and compared to find the best model according to the AIC criterion. Different random-effect combinations were tested: country alone, cluster alone, cluster and country, and cluster nested in country. For Global Acute Malnutrition and Moderate Acute Malnutrition (WHZ, MUAC and indicators combined), the best model was the one with cluster nested in country. For Severe Acute Malnutrition, the best model was the one with cluster alone. Cluster: the smallest geographic units in each country. WHZ: Weight for Height Z-score; MUAC: Mid-upper Arm Circumference. cGAM: combined Global Acute Malnutrition; cMAM: combined Moderate Acute Malnutrition; cSAM: combined Severe Acute Malnutrition. Std. Dev.: Standard deviation. * Conditional modes of the random effects.
(DOCX)
