# Supplementary material for: Factors associated with acute malnutrition among children aged 6–59 months in Haiti, Burkina Faso and Madagascar: A pooled analysis
Source: PLoS One. 2022 Dec 12;17(12):e0278980. doi: 10.1371/journal.pone.0278980 (PMC9744306; doi:10.1371/journal.pone.0278980)
Supplement: S1 Appendix — (PDF) [file pone.0278980.s001.pdf]

ENQUETE NUTRITIONNELLE NATIONALE 2018 VISA : AP2010004CNSCS2

Questionnaire ménage pour le donneur de soin principal (mère)

Date enquête : _____/_____/ <u>**2018**</u> **Equipe**: /____/____/ ____/ **Chef d'équipe**: ___________________________________

Grappe (ZD) :________________________________/___/___/___/___/___/

Région : ____________________________**Province**:_____________________________

N° du ménage : /___/___/___/

1.Quelle est la taille du ménage?	Nombre d'individus ______________	
2.Combien d'enfants âgés de 0 à 59 mois font partis de ce ménage ?	Nombre d'enfants ______________	
3.Quel est le sexe du chef du ménage?	Homme ……………………………………………<br>Femme ……………………………………………	1<br>0
4.Quel est le statut marital du chef de ménage ?	Marié monogame ……………………………<br>Marié polygame ………………………………<br>Veuf / Veuve ……………………………………<br>Séparé ou divorcé ……………………………	0<br>1<br>2<br>3
5.Lors de ces douze derniers mois, existait-il des mois pendant lesquels vous n'aviez pas assez de nourriture pour satisfaire les besoins de votre famille ?	Oui ……………………………………………………<br>Non ……………………………………………………	1<br>0
6.Si la réponse est **OUI,** quels étaient les mois (lors de ces douze derniers mois) pendant lesquels vous n'aviez pas assez de nourriture pour répondre aux besoins de votre famille ?<br><br>Il faut inclure tout type de nourriture peu importe d'ailleurs la source de la nourriture, telle que production, achat ou troc, aide alimentaire ou emprunts.<br><br>**Ne pas lire à voix haute la liste des mois.** Merci de mettre 1 dans la case si la personne interrogée identifie ce mois comme un mois pendant lequel le ménage n'avait pas assez de nourriture pour répondre à leurs besoins. Si la personne interrogée n'identifie pas ce mois, merci de mettre 0 dans la case.<br><br>En cas de besoin veuillez utiliser un calendrier indiquant les saisons afin d'aider la personne	Oui 1 Non 0	
	Septembre 2018	|__|
	Aout 2018	|__|
	Juillet 2018	|__|
	Juin 2018	|__|
	Mai 2018	|__|
	Avril 2018	|__|
	Mars 2018	|__|
	Février 2018	|__|
	Janvier 2018	|__|
	Décembre 2017	|__|
	Novembre 2017	|__|
	Octobre 2017	|__|

interrogée à se souvenir des différents mois.		
7. Lorsque que vous avez un problème, vous sentez-vous supportée/ aidée/ entourée ? (Par votre famille, la communauté, vos amis...)	Extrêmement ……………………………………<br>Plutôt supportée ………………………………<br>Pas vraiment ……………………………………<br>Pas du tout ………………………………………	3<br>2<br>1<br>0
8.Combien d'animaux possédez-vous dans le ménage ?<br>**Ecrivez le nombre d'animaux pour chaque catégorie (de 0 à …………)**		Nombre
	81Vache, bœufs ?	
	82Chèvres, moutons	
	83Poules ?	
	84Chevaux ?	
	85Anes, mules ?	
	86Porc, cochons ?	
	Autres, préciser : ______________<br>____________________________	
9.Combien de temps mettez-vous pour aller au point d'eau, puiser l'eau et revenir ?<br>**Entourer la réponse donnée**	1. Moins de 30 minutes<br>2. Entre 30 et 60 minutes<br>3. Plus de 60 minutes	
10.Combien de temps mettez-vous pour aller jusqu'au centre de santé le plus proche ?<br>**Entourer la réponse donnée**	1. Moins de 30 minutes<br>2. Entre 30 et 60 minutes<br>3. Plus de 60 minutes	
11.Quel est le type de sources d'eau que vous utilisez pour l'eau de boisson en ce moment ?<br>**Entourer la ou les réponses données**	1. forages équipés d'une pompe à main, puits ou forages équipés d'un système de pompe motorisé<br>2. puits ou forage ouvert<br>3. Système de collecte des eaux de pluie<br>4. Distribution par réseau<br>5. Source ouverte non protégée<br>6. Autre	
12.Dans votre ménage qui décide comment dépenser l'argent du ménage que vous gagnez?<br>**Entourer la ou les réponses données**	Vous ………………………………………………<br>Le père …………………………………………<br>Les deux …………………………………………	4<br>3<br>2

	Quelqu'un d'autre………………………………	1
13.Qui décide si l'enfant doit aller à l'école ?<br><br>**Entourer la ou les réponses données**	Vous………………………………………………<br>Le père………………………………………………<br>Les deux ……………………………………………<br>Quelqu'un d'autre ……………………………	4<br>3<br>2<br>1
14.Qui prend les décisions concernant les soins de santé de l'enfant pour l'emmener au Centre de santé ?<br><br>**Entourer la ou les réponses données**	Vous………………………………………………<br>Le père………………………………………………<br>Les deux ……………………………………………<br>Quelqu'un d'autre ……………………………	4<br>3<br>2<br>1
15. Où s'est déroulé votre dernier accouchement?	Au centre de santé ……………………………<br>A la maison ………………………………………<br>Autre précisez : ___________________	1<br>0
16. Que faites-vous d'habitude pour traiter l'eau de boisson? (ne pas citer)<br><br>**Entourer la ou les réponses données**	Porter l'eau à ébullition ………………………<br>Ajout de chlore …………………………………<br>Filtrer à travers un tissu ……………………<br>Utilisation d'un filtre à eau ………………<br>Désinfection solaire……………………………<br>Laisser reposer le dépôt ……………………<br>Autre, Préciser ___________________<br>Ne sait pas …………………………………………	1<br>2<br>3<br>4<br>5<br>6<br>7<br>99
17. Est-ce qu'actuellement vous avez du savon dans le ménage ?	Oui ……………………………………………………<br>Non………………………………………………………	1<br>0
18. A quel âge avez-vous eu votre premier enfant ?	Age en année ________________________<br>Ne sait pas …………………………………………	 99
19. Avez-vous pu vous reposer après vous dernier accouchement ? (pas de travail)	Non ……………………………………………………<br>Oui, Moins de 40 jours ………………………<br>Oui, Plus de 40 jours …………………………	0<br>1<br>2
20. Pour votre dernière grossesse avez-vous été aux consultations prénatales ?	Oui ……………………………………………………<br>Non ……………………………………………………	1<br>0
21. Combien de fois avez-vous été aux consultations prénatales durant votre dernière grossesse ?	Nombre ________________________	

22 Observation de l'enquêteur : Y a-t-il des ustensiles de cuisine ou pour boire laissés sur le sol ?	Oui ……………………………………………………<br>Non……………………………………………………	1<br>0
23. Observation de l'enquêteur : Y a-t-il de la nourriture laissée à découvert ?	Oui ……………………………………………………<br>Non ……………………………………………………	1<br>0
24. Est-ce que des animaux ont accès à l'intérieur de la maison et/ ou dans la cuisine ?	Oui ……………………………………………………<br>Non ……………………………………………………	1<br>0
25. Voyez-vous des déchets organiques d'animaux ou d'humain à proximité (5 à 10 mètres) de la cuisine ?	Oui ……………………………………………………<br>Non ……………………………………………………	1<br>0
26. Le récipient de stockage de l'eau de boisson est-il ouvert ou fermé ?	Ouvert………………………………………………<br>Fermé………………………………………………	0<br>1
27. Le récipient utilisé pour le stockage de l'eau de boisson est-il le même que celui utilisé pour le transport ?	Oui……………………………………………………<br>Non ……………………………………………………	1<br>0
28. Observation : y-a-t-il des latrines dans le ménage ?	Oui 1<br>Non 0	

ENQUETE NUTRITIONNELLE NATIONALE 2018 VISA : AP2010004CNSCS2

Questionnaire enfants 0-59 mois (A administrer à chaque enfant entre 0 et 59 mois en plus des questionnaires de la SMART nationale)

Date enquête : _____/_____/ 2018 **Equipe**: /____/____/ ____/ **Chef d'équipe**: ___________________________________

Grappe (ZD) :_________________________________/___/___/___/___/

Région : ____________________________**Province**:_____________________________

N° du ménage : /___/___/___/

1. Quel est le lien avec cet enfant ?<br><br>**Entourer la réponse donnée**	1. La mère<br>2. La grand-mère<br>3. Autres, préciser : ______________	
2. Est-ce que (nom) a eu la diarrhée au cours des deux dernières semaines? (plus de 3 selles molles ou liquides dans un délai de 24 heures)	Oui…………………….. ..<br>Non………………………	1<br>0
3. Si **Oui,** avez-vous recherché un traitement à ce moment-là au centre de santé ?	Oui ………………………<br>Non ………………………	1<br>0
4. Est-ce que l'enfant a un petit frère ou une petite sœur?	Oui ………………………<br>Non ………………………	1<br>0
5. Quel est l'écart d'âge avec son/ sa petite sœur frère direct?	Plus de 24 mois………<br>Moins de 24 mois…..	1<br>0
6. Est-ce qu'il arrive que l'enfant soit gardé par un autre enfant (de moins de 12 ans) lorsque vous êtes absente ?	Oui………………………<br>Non………………………	1<br>0
7. Quel était le poids de naissance de l'enfant ?<br>(vérifier sur un certificat)	Poids en kg : ______<br>Ne sait pas……………	 999
Observation de l'enquêteur sur l'endroit où est l'enfant pendant l'entretien :<br><br>8. Y a-t-il des animaux présents dans cette zone ?<br><br>9. Y a-t-il des excréments d'animaux ou d'autres signes de présence animale (par exemple des plumes) visibles dans les zones identifiées ?	 <br>Oui………………………<br>Non………………………<br><br>Oui………………………<br>Non ………………………	 <br>1<br>0<br><br>1<br>0
Observation de l'enquêteur sur la propreté de l'enfant :<br><br>10. L'enfant a-t-il le visage et les mains propres ?	 <br>Oui………………………<br>Non………………………	 <br>1<br>0

11. L’enfant a-t-il des habits propres ?	Oui……………………….<br>Non………………………..	1<br>0
12. L’enfant semble-t-il récemment lavé ?	Oui……………………….<br>Non………………………..	1<br>0

ENQUETE NUTRITIONNELLE NATIONALE 2018 **VISA : AP2010004CNSCS2**

Diversité alimentaire Femmes

Date enquête :_____/_____/**2018** **Equipe**: /___/___/___/ **Chef d'équipe**: ______________________

Grappe (ZD) :____________________/___/___/___/ ___/ **Région** : ________________ **Province**:____________

N° du ménage : /___/___/___/

K000 Nombre Femme en Age de Procréer (15-49 ans) dans le ménage ☐

Consultez la table de Kish et notez le no. de ligne du FAP 15-49 ans sélectionnée dans la case

N° d'ordre	Nom & prénom	Age
1		
2		
3		
4		
5		
6		
7		
8		
9		
10		

[illegible] ☐

QD00. Est-ce que, pendant la journée d'hier, vous avez mangé de façon inhabituelle en comparaison avec les autres jours de la semaine? (fête, cérémonie, ou à l'inverse absence inhabituelle de repas) **OUI = 1 ; NON = 2** |__|

Si la réponse est "oui", posez la même question pour le jour surpassé ; renseigner le tableau avec la consommation des jours ordinaires.

*Nous nous intéressons à tout ce que vous avez mangé ou bu dans la **journée d'hier**, entre le moment de votre réveil hier matin et celui de votre réveil ce matin.*

Reveil/petit-déjeuner	matinée	repas de midi	après-midi	repas du soir	soirée/nuit

Maintenant, je voudrais vous demander de décrire tout ce que vous avez mangé ou bu hier durant le jour ou la nuit, que vous l'ayez mangé à la maison ou ailleurs. Veuillez inclure tous les aliments et boissons, les collations ou les petits repas, ainsi que les repas principaux.

NO.	GROUPES D'ALIMENTS	EXEMPLES	CONSOMMATION OUI = 1 NON = 2
QD01	CEREALES	1.Sorgho, 2.mil (petit mil, brisure, farine), 3.maïs (brisure ou farine), 4.maïs grillé doux, 5.fonio, 6.riz, 7.pâtes alimentaires (macaronis, etc.), 8.blé, pain…9. Autre (préciser)_____________	|__|
QD02	RACINES ET TUBERCULES	1.Patate douce blanche, 2.pomme de terre,3. manioc, 4.banane plantain (aloco), 5. Ignames 6.Autre(préciser)_____________	|__|
QD03	LEGUMINEUSES	1.Haricots (niébé), 2.pois de terre/woandzou, 3.pois chiches, 4.lentilles, 5.autres légumes secs, 6.sésame, 7.pois sucrés, 8.soja et produits dérivés, 9.Autre(préciser)_____________	|__|
QD04	NOIX ET GRAINES	1.graine de nénuphar, 2. arachide (en pâte ou nature, tourteaux), 3.noix sauvages, 4.datou ou soumbala, bikalga (graine d'oseille fermentées) 5.Autre(préciser)_____________	|__|
QD05	LAIT ET PRODUITS LAITIERS	1.Lait frais, 2.lait en poudre, 3.lait concentré (sucré ou non), 4.lait caillé naturel, 5.yaourt, 6.fromages 6.Autre(préciser)_____________	|__|
QD06	ABATS	1.Foie, 2.reins, 3.cœur, 4.poumons, ou tout autre abat (de veau, de mouton, de chèvre, de volailles), 5.viscères (soupe de viscères) 6.Autre(préciser)_____________	|__|
QD07	VIANDES ET PRODUITS CARNES	1.Bœuf, 2.mouton, 3.chèvre, 4.lapin, 5.poulet, 6.pintades, 6.oiseaux, 7.canard viande de brousse (gazelle, varan, tortue, rats sauvages, agoutis, écureuils, perdrix, serpent, souris, biches…°) 8.Autre(préciser)_____________	|__|
QD08	POISSONS ET FRUITS DE MER	1.Poisson frais, 2.poisson fumé, salé, conserves (sardines, thon...), 3.crevettes, 4.escagot, 5.crabe 6.Autre(préciser)_____________	|__|
QD09	ŒUFS	1.Œufs de poule,2. de pintade, 3.de canard, 4.caille, 5.oie… 6.Autre(préciser)_____________	|__|
QD10	LEGUMES FEUILLES VERTES FONCEES	1.feuilles de baobab, 2.feuilles d'échalote verte foncée,3. feuilles d'oignon frais, 4.feuilles de jaxatou (Yalo), 5.toutes feuilles sauvages vertes foncées 6.Autre (préciser)_____________	|__|
QD11	RACINES, TUBERCULES ET LEGUMES RICHES EN VIT A	1.Patate douce à chair orangée, 2.Feuilles d'oseille (dah), 3.feuilles de courge, 4.feuilles de haricot, 5.feuilles de moringa, 6.feuilles de patates, 7.feuilles de manioc, 9.Autre (préciser)_____________	|__|
QD12	FRUITS RICHE EN VITAMINE A	1.Mangue, 2.papaye, 3.citrouille, 4.courge, 5.melon à chair orange, 6. néré/poudre de néré…7.Autre(préciser)_____________	|__|
QD13	AUTRES LEGUMES	1.Tomates fraîches, 2. gombo frais ou sec, 3.aubergines, 4.aubergines locales (jaxatus ou yalo), 5.courgettes, 6.concombres, 7.choux, 8.oignons, 9.échalote fraîche, 10.poivrons verts, 11.haricots verts, 12.laitue (salade), 13.épinard, 14.Autre (préciser)_____________	|__|
QD14	AUTRES FRUITS	1.Banane, 2.goyave, 3.pastèque, 4.orange, 5.citron, 6.dattes, 7.jujube, 8.fruits sauvages ("raisin"/bembé, pain de singe/fruit de baobab), 9.« dattes » sauvages (Aduwa), 10.pulpe de karité, 11.fruit de liane, 12.chair de fruit de rônier, 13.ananas, avocats…) 14.Jus de fruits frais (fruits pressés) 15.Autre(préciser)_____________	|__|

**QD15**	**HUILE DE PALME ROUGE**	1. Huile de palme rouge, 2. aliments préparés avec de la noix de palme rouge ou de la pulpe de noix de palme rouge 3.Autre(préciser)_____________	|__|
**QD16**	**INSECTES**	1. Insectes, 2.chenille, 3.les œufs d'insectes, 4. Criquets/sauterelles 5. Ephémères<br><br>5.Autre (préciser)_____________	|__|
**QD17**	**HUILE ET GRAISSE**	1.Huile végétale (d'arachide, de sésame, de coco, etc. - pour sauces, assaisonnements, fritures...), 2.beurre/huile de karité, 3.beurre de vache (sirimè), 4.graisses végétales/margarine, 5.mayonnaise, 6.graisses animales…7.Autre(préciser)_____________	|__|
**QD18**	**SUCRE ET PRODUITS SUCRES**	1.Sucre en poudre ou en morceaux (dans le thé, le café, la bouillie...), 2.boissons sucrées (boisson gazeuse/sucrerie, bissap, jus de gingembre, jus de feuilles ou de fruits de tamarin sucré, citronnelle), 3. miel, 4.confiture, 5.bonbons, 6.beignets sucrés, 7.galettes sucrées, 8.biscuits sucrés, 9.Thé, 10.café, 11.chikoré, 12.kinkeliba, 13.jus de feuille ou de fruit de tamarin non sucré 14..Autre(préciser)_____________	|__|
**QD19**	**CONDIMENTS, EPICES**	1. piment, 2.poivre, 3.vinaigre, 4.ail, 5.sachet d'épices, 6.sel, 7.cube Maggi, 8. Maggi blanc, 9.laurier, 10.tomate concentrée, 11.condiment à base d'oseille (graine/datou, feuilles ou pulpe,) 12.condiment à base d'oignon ou de feuilles d'oignon séchée/transformée ou 13.condiment à base de soja <u>14. Petite quantité</u> de poudre de poisson, 15.de poudre de gombo, 16.de feuille de baobab sèche, 17.de poivron, 18.de soumbala 17.Autre(préciser)_____________	|__|
