# Supplementary figures and images for: Factors associated with acute malnutrition among children aged 6–59 months in Haiti, Burkina Faso and Madagascar: A pooled analysis
Source: PLoS One. 2022 Dec 12;17(12):e0278980. doi: 10.1371/journal.pone.0278980 (PMC9744306; doi:10.1371/journal.pone.0278980)

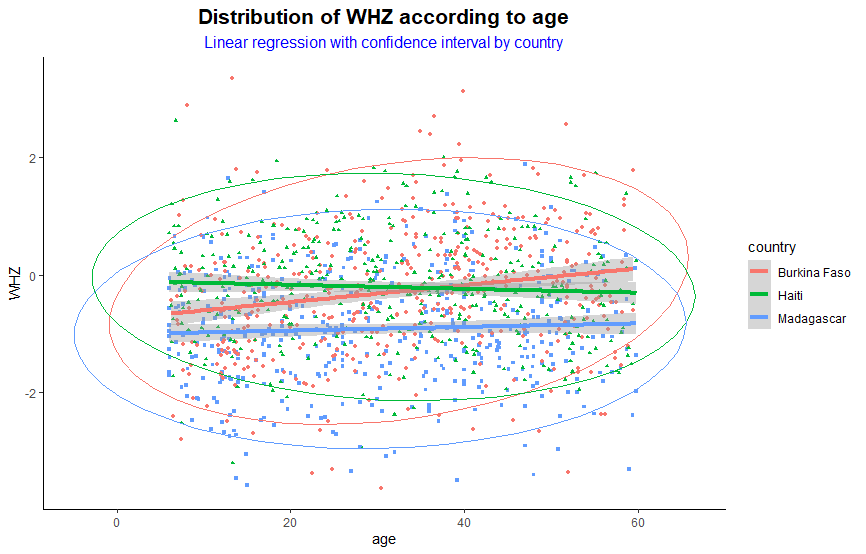

Supplement: S1 Fig — WHZ: Weight for Height Z-score; age in months. (TIF) [file pone.0278980.s002.tif]

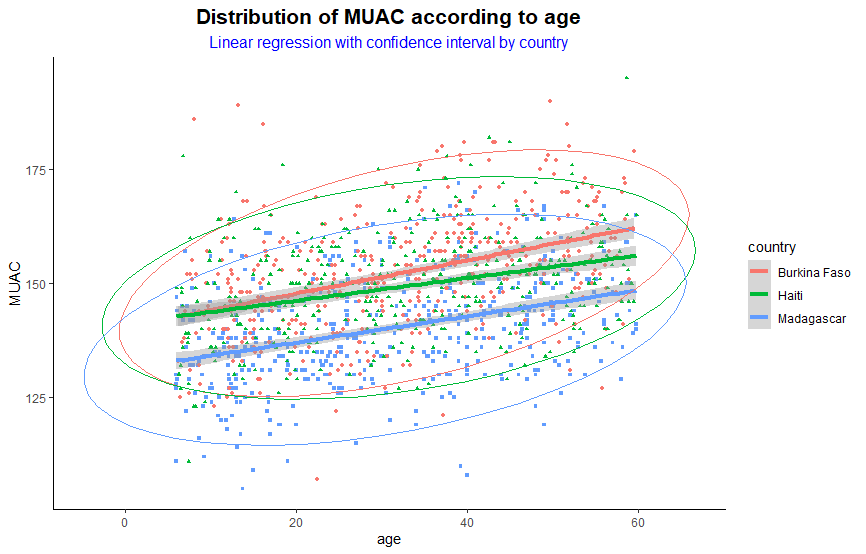

Supplement: S2 Fig — MUAC: Mid-upper Arm Circumference; age in months. (TIF) [file pone.0278980.s003.tif]
