# Supplementary material for: Factors associated with acute malnutrition among children aged 6–59 months in Haiti, Burkina Faso and Madagascar: A pooled analysis
Source: PLoS One. 2022 Dec 12;17(12):e0278980. doi: 10.1371/journal.pone.0278980 (PMC9744306; doi:10.1371/journal.pone.0278980)
Supplement: S1 Table — (DOCX) [file pone.0278980.s004.docx]

**S1 Table. Study areas and populations**

|  | **Burkina Faso** | **Madagascar** | **Haiti** |
| --- | --- | --- | --- |
| **Study area** | Province of Kénédougou in the region of the Haut-Bassins | District of Amboasary Sud in the Anosy region | The communes of Anse d’Hainault, Dame Marie and Les Irois in The Grande Anse region |
| **Area** | 8,307 km² | 10,211 km² | 326.52 km² |
| **Population** | More than 280,000 inhabitants | 300,775 inhabitants | 98,522 inhabitants |
| **Population density** | 35 inhabitants per km² | 30 inhabitants per km² | 302 inhabitants per km² |
| **Age distribution** | Young population, with 48% of the population is under 14 years old and just 3% is over 65 years old | 60% of the population is under 20 years of age | 41% of them are less than 18 years old |
| **Composition of study area** | The Province of Kénédougou is composed of 13 departments, each composed of villages and 92% of the population lives in rural areas | According to the Malagasy classification, Amboasary Sud encompasses several livelihood zones [1].  The district consists of 18 communes subdivided into 303 fokontany (villages), which are further subdivided into hamlets [2] | The commune of Anse d’Hainault is composed of 4 the communal sections.  The commune Dame Marie is composed of 6 communal sections.  Les Irois is composed of 3 communal sections |
| **Climate** | High rainfall (more than 1000 mm annually) and rich and diversified soils. The region’s heavy rainfall and flooding strain household livelihoods based primarily on agriculture and livestock farming, especially for the most vulnerable populations [3] | Rainy season (November to March) and a dry season (April to October) with an annual average rainfall of 924 mm | The department benefits from a varied climate. Precipitation is much greater in summer (April to October) than in winter (December to February) with an annual average rainfall of 608 mm |
| **Agriculture and economy** | The poverty rate is 47.8% and a minority of the population has access to potable water and adequate sanitation [4].  The primary agricultural products include cereals, tubers, fruits, vegetables, cotton and cattle are the main livestock raised for market. | The zone MG22^1^ produces rice and onion, the zone MG24 provides mainly cassava, maize, sweet potato and livestock, and the zone MG26 is known for the cassava and maize production as well as livestock breeding [5]. | The economy of these three communes is based on agriculture, particularly cocoa and coffee production, fishing and livestock farming. |
| **Ethnic composition** | More than 30 ethnic groups:   - Senoufo (54%) - Samogo (10%) - Sèmè (6%) | Four different ethnic groups:   - The Antandroy who are a traditionally nomadic ethnic group who heard cattle for a living (mainly zebus) - The Antanosy who are mainly fishermen - The Betsileos who are rice farmers - The Bara who are distinctly zebu and goat herders, often migrating in search of new pastures [2] | - Mostly African descent, (with 5% considered as mixed African and other ancestry) [6] - Indigenous Taíno, though, people of various ethnic backgrounds have settled in the country |
| **Global Acute Malnutrition(GAM) prevalence** | In 2016, GAM by Weight for Height Z-score (WHZ) prevalence was 7.8% [7] | In 2018, GAM by WHZ prevalence was 12.9% [2] | The prevalence of GAM by WHZ was 5% [8] |

^1^ Rural livelihood zone defined as a geographical area where households share the same ecology and systems of production, the same choices of suitable crops and livestock, the same market conditions, and the same options for generating cash income [5]. The three regions mentioned are included in our study area.

**REFERENCES**

[1] *Rapport final : Link NCA nutrition causal analysis - Madagascar*. Madagascar: Action Contre la Faim, http://linknca.org/etude/district_of_amboasary_anosy_region.htm (2019, accessed 12 June 2020).

[2] *Enquête nutritionnelle et de mortalité rétrospective, District d’Amboasary Atsimo, Madagascar, 2018*. Madagascar, 2018.

[3] Food and Agriculture Organization of the United Nations. *The State of Food and Agriculture 2015: Social Protection and Agriculture - Breaking the Cycle of Rural Poverty*. UN. Epub ahead of print 15 May 2015. DOI: 10.18356/88b78e6f-en.

[4] Yemdaogo Z, Honkounne KS. CARTOGRAPHIE DE LA PAUVRETE ET DES INEGALITES AU BURKINA FASO. 87.

[5] *MADAGASCAR Grand South livelihood zones revision*. Famine Early Warning Systems Network, https://fews.net/sites/default/files/documents/reports/Madagascar_Grand_South_Livelihood_Zoning_Revision.pdf (November 2017, accessed 20 July 2022).

[6] Estimation de la Population 2015 - IHSI. *pdfhall.com*, https://pdfhall.com/estimation-de-la-population-2015-ihsi_59fd10ca1723dd743b185fdf.html (accessed 22 April 2020).

[7] *Enquête nutritionnelle nationale SMART 2016*. Burkina Faso: Ministère de la Santé, 2016.

[8] *Enquête Mortalité, Morbidité et Utilisation des Services (EMMUS-VI 2016-2017 )*. Haiti: Institut Haïtien de l’Enfance, https://www.unicef.org/haiti/rapports/emmus-vi-rapport-de-synth%C3%A8se-2018 (2018, accessed 12 June 2020).
