# Supplementary material for: Factors associated with acute malnutrition among children aged 6–59 months in Haiti, Burkina Faso and Madagascar: A pooled analysis
Source: PLoS One. 2022 Dec 12;17(12):e0278980. doi: 10.1371/journal.pone.0278980 (PMC9744306; doi:10.1371/journal.pone.0278980)
Supplement: S2 Table — (DOCX) [file pone.0278980.s005.docx]

**S2 Table. Parameters used to calculate the sample size.**

|  | Estimated Prevalence (GAM) | Desired precision | Design effect | Percent of non-respondent | Average household size | Percent of under five children |
| --- | --- | --- | --- | --- | --- | --- |
| Burkina Faso | 11.6% | 3.5% | 1.5 | 2% | 6.0 | 17.8% |
| Madagascar | 12.9% | 3.5% | 1.3 | 5% | 5.3 | 19.2% |
| Haiti | 8.6% | 4% | 1.5 | 3% | 4.8 | 12.8% |
