# Supplementary material for: Factors associated with acute malnutrition among children aged 6–59 months in Haiti, Burkina Faso and Madagascar: A pooled analysis
Source: PLoS One. 2022 Dec 12;17(12):e0278980. doi: 10.1371/journal.pone.0278980 (PMC9744306; doi:10.1371/journal.pone.0278980)
Supplement: S3 Table — * Recoding as “1” corresponds to unprotected main drinking water source and “0” corresponds to protected main drinking water source. (DOCX) [file pone.0278980.s006.docx]

**S3 Table. Recoding of the variable main drinking water source**

| **Madagascar** | | | **Burkina Faso** | | | **Haiti** | | |
| --- | --- | --- | --- | --- | --- | --- | --- | --- |
|  | Recoding* |  | | Recoding* |  | | Recoding* |  |
| Source (protected or unprotected) | 1 | Open source unprotected | | 1 | Open source | | 1 |  |
| Wells « Vovo » | 1 | Open well or drilling | | 1 | Large diameter well | | 1 |  |
| River, lake, canal | 1 | - | | - | River, ravin, canal | | 1 |  |
| Catchment basin or impluvium (rainwater) | 0 | Rainwater collecting system | | 0 | Rainwater | | 0 |  |
| Tap | 0 | Distribution by network | | 0 | Pipe | | 0 |  |
| Drilling or pumped well | 0 | Boreholes equipped with a hand pump, wells or boreholes equipped with a motorized pump system | | 0 | Tank or water castle. | | 0 |  |
| - | - | - | | - | Reservoir | | 0 |  |
| - | - | - | | - | Artesian well | | 0 |  |

* Recoding as “1” corresponds to unprotected main drinking water source and “0” corresponds to protected main drinking water source.
