# Supplementary material for: Factors associated with acute malnutrition among children aged 6–59 months in Haiti, Burkina Faso and Madagascar: A pooled analysis
Source: PLoS One. 2022 Dec 12;17(12):e0278980. doi: 10.1371/journal.pone.0278980 (PMC9744306; doi:10.1371/journal.pone.0278980)
Supplement: S4 Table — ***p-value < 0.001; **p-value < 0.01; *p-value < 0.05; ●p-value < 0.20; Ref: the reference class for a qualitative variable; OR: Odds ratio; CI 95%: confidence interval at 5% alpha risk; WHZ: Weight for Height Z-score; MUAC: Mid-upper Arm Circumference; GAM: Global Acute Malnutrition; MAM: Moderate Acute Malnutrition; SAM: Severe Acute Malnutrition; cGAM: combined Global Acute Malnutrition; cMAM: combined Moderate Acute Malnutrition; cSAM: combined Severe Acute Malnutrition. (DOCX) [file pone.0278980.s007.docx]

**S4 Table. Univariate models between anthropometric indicators and risk factors for malnutrition**

|  |  | WHZ | | | | | | MUAC | | | | | | combined WHZ and MUAC | | | | | |
| --- | --- | --- | --- | --- | --- | --- | --- | --- | --- | --- | --- | --- | --- | --- | --- | --- | --- | --- | --- |
|  |  |  |  |  |  |  |  |  |  |  |  |  |  |  |  |  |  |  |  |
|  |  | GAM | | MAM | | SAM | | GAM | | MAM | | SAM | | cGAM | | cMAM | | cSAM | |
|  |  | OR | CI 95% | OR | CI 95% | OR | CI 95% | OR | CI 95% | OR | CI 95% | OR | CI 95% | OR | CI 95% | CI 95% | IC 95% | OR | CI 95% |
| Sex of the child - | *Female (ref)* | 1.0 |  | 1.0 |  | 1.0 |  | 1.0 |  | 1.0 |  | 1.0 |  | 1.0 |  | 1.0 |  | 1.0 |  |
|  | *Male* | **1.6** | **(1.1-2.6)*** | **2.0** | **(1.2-3.3)**** | 0.7 | (0.2-1.8) | 0.5 | (0.3-1.0) ● | 0.8 | (0.4-1.7) | **0.1** | **(0.0-0.7)*** | 1.4 | (0.9-2.1)● | **1.6** | **(1.0-2.4)*** | 0.6 | (0.2-1.4) |
| Vitamine A - | *Yes (ref)* | 1.0 |  | 1.0 |  | 1.0 |  | 1.0 |  | 1.0 |  | 1.0 |  | 1.0 |  | 1.0 |  | 1.0 |  |
|  | *No* | 1.2 | (0.7-2.0) | 0.8 | (0.4-1.6) | 3.0 | (1.0-9.5)* | **3.2** | **(1.5-6.8)**** | 2.1 | (0.9-5.0)● | **20.6** | **(2.3-182.3)***** | 1.5 | (0.9-2.5)● | 1.2 | (0.7-2.2) | **5,0** | **(1.7-15.0)***** |
| Deworming - | *Yes (ref)* | 1.0 |  | 1.0 |  | 1.0 |  | 1.0 |  | 1.0 |  | 1.0 |  | 1.0 |  | 1.0 |  | 1.0 |  |
|  | *No* | 1.4 | (0.8-2.4) | 1.3 | (0.7-2.3) | 1.6 | (0.5-5.1) | **3,0** | **(1.2-7.3)*** | 2.5 | (0.9-7.3) ● | 3.7 | (0.5-29.0) | 1.5 | (0.9-2.5)● | 1.5 | (0.9-2.6)● | 2.1 | (0.6-6.8) |
| Diarrhea per two-week recall | *No (ref)* | 1.0 |  | 1.0 |  | 1.0 |  | 1.0 |  | 1.0 |  | 1.0 |  | 1.0 |  | 1.0 |  | 1.0 |  |
|  | *Yes* | 1.6 | (1.0-2.5) ● | **2.0** | **(1.2-3.3)***** | 0.5 | (0.1-1.9) | **2.9** | **(1.5-5.9)***** | **4,0** | **(1.8-8.9)***** | 1.1 | (0.2-5.6) | **1.8** | **(1.2-2.8)***** | **2.1** | **(1.3-3.3)***** | 0.6 | (0.2-1.8) |
| Fever per two-week recall | *No (ref)* | 1.0 |  | 1.0 |  | 1.0 |  | 1.0 |  | 1.0 |  | 1.0 |  | 1.0 |  | 1.0 |  | 1.0 |  |
|  | *Yes* | **1.6** | **(1.0-2.5)*** | **2.2** | **(1.3-3.7)***** | 0.3 | (0.1-1.2)● | 1.5 | (0.7-2.9) | 1.6 | (0.7-3.5) | 0.9 | (0.1-5.9) | 1.5 | (1.0-2.3) ● | **1.7** | **(1.1-2.7)*** | 0.7 | (0.2-1.9) |
| Clean face and hands | *Yes (ref)* | 1.0 |  | 1.0 |  | 1.0 |  | 1.0 |  | 1.0 |  | 1.0 |  | 1.0 |  | 1.0 |  | 1.0 |  |
|  | *No* | 1.6 | (1.0-2.8) ● | 1.7 | (1.0-3.1) ● | 1.6 | (0.5-4.6) | 2.1 | (0.9-4.8) ● | 1.9 | (0.7-5.2)● | 2.1 | (0.3-15.0) | 1.5 | (0.9-2.4)● | 1.5 | (0.9-2.6)● | 1.6 | (0.5-5.0) |
| Clean clothes - | *Yes (ref)* | 1.0 |  | 1.0 |  | 1.0 |  | 1.0 |  | 1.0 |  | 1.0 |  | 1.0 |  | 1.0 |  | 1.0 |  |
|  | *No* | 1.2 | (0.7-2.1) | 1.3 | (0.7-2.5) | 1.2 | (0.4-3.4) | 2,0 | (0.8-5.2)● | 2.2 | (0.7-6.8)● | 1.7 | (0.2-12.8) | 1.2 | (0.7-2.0) | 1.3 | (0.8-2.4) | 1.2 | (0.4-3.9) |
| Female head of household - | *No (ref)* | 1.0 |  | 1.0 |  | 1.0 |  | 1.0 |  | 1.0 |  | 1.0 |  | 1.0 |  | 1.0 |  | 1.0 |  |
|  | *Yes* | 1.1 | (0.7-1.8) | 1.2 | (0.7-2.0) | 0.8 | (0.2-2.4) | 1.0 | (0.5-2.0) | 0.9 | (0.4-2.1) | 2.3 | (0.6-9.1) | 1.0 | (0.6-1.6) | 1,0 | (0.6-1.7) | 0.8 | (0.3-2.3) |
| Large family (>country average) - | *No (ref)* | 1.0 |  | 1.0 |  | 1.0 |  | 1.0 |  | 1.0 |  | 1.0 |  | 1.0 |  | 1.0 |  | 1.0 |  |
|  | *Yes* | **0.6** | **(0.4-0.9)*** | 0.6 | (0.4-1.0) ● | 0.4 | (0.1-1.1) ● | **0.3** | **(0.1-0.6)**** | **0.2** | **(0.1-0.7)**** | 0.1 | (0.0-1.0) ● | **0.5** | **(0.3-0.7)**** | **0.5** | **(0.3-0.8)**** | 0.4 | (0.1-1.1) ● |
| Inadquate household food provisioning per 12-month recall | *No (ref)* | 1.0 |  | 1.0 |  | 1.0 |  | 1.0 |  | 1.0 |  | 1.0 |  | 1.0 |  | 1.0 |  | 1.0 |  |
|  | *Yes* | 0.7 | (0.3-1.4) | 0.7 | (0.3-1.7) | 0.9 | (0.2-3.2) | 1.9 | (0.5-7.3) | 2.0 | (0.4-9.4) | 2.2 | (0.1-47.5) | 0.8 | (0.4-1.7) | 0.9 | (0.4-2.1) | 1.2 | (0.3-4.3) |
| Water source - | *Protected (ref)* | 1.0 |  | 1.0 |  | 1.0 |  | 1.0 |  | 1.0 |  | 1.0 |  | 1.0 |  | 1.0 |  | 1.0 |  |
|  | *No protected* | 0.7 | (0.4-1.1)● | 0.6 | (0.4-1.1)● | 0.9 | (0.3-2.5) | 1.3 | (0.5-3.1) | 1.4 | (0.5-4.2) | 1.7 | (0.2-16.8) | 0.7 | (0.4-1.2) | 0.7 | (0.4-1.2)● | 1.3 | (0.4-4.4) |
| Birth location - | *Out of home (ref)* | 1.0 |  | 1.0 |  | 1.0 |  | 1.0 |  | 1.0 |  | 1.0 |  | 1.0 |  | 1.0 |  | 1.0 |  |
|  | *Home* | 1.0 | (0.5-1.8) | -0.2 | (-0.8-0.5) | 1.8 | (0.5-7.0) | 1.2 | (0.5-2.8) | 1,0 | (0.4-2.6) | 2.1 | (0.4-10.5) | 1.0 | (0.6-1.7) | 0.9 | (0.5-1.7) | 1.4 | (0.4-4.9) |
| pre-natal consultation < 4 - | *No (ref)* | 1.0 |  | 1.0 |  | 1.0 |  | 1.0 |  | 1.0 |  | 1.0 |  | 1.0 |  | 1.0 |  | 1.0 |  |
|  | *Yes* | 1.4 | (0.8-2.3) | 1.5 | (0.9-2.7)● | 0.8 | (0.2-3.1) | 1.3 | (0.5-3.0) | 0.8 | (0.3-2.1) | 3.7 | (0.7-20.3)● | 1.5 | (0.9-2.5)● | 1.5 | (0.9-2.6)● | 1.2 | (0.3-4.1) |

*****p-value < 0.001; **p-value < 0.01;*p-value < 0.05**; ●p-value < 0.20;

Ref : the reference class for a qualitative variable;

OR : Odds ratio; CI 95%: confidence interval at 5% alpha risk;

WHZ: Weight for Height Z-score;

MUAC: Mid-upper Arm Circumference;

MAM: Moderate Acute Malnutrition (for WHZ: -3≤WHZ<-2; For MUAC: 115≤MUAC<125 mm);

SAM: Severe Acute Malnutrition (for WHZ: WHZ <-3 and/or presence oedema; For MUAC: MUAC <115 mm and/or presence oedema);

GAM: Global Acute Malnutrition (SAM or MAM);

cGAM: combined Global Acute Malnutrition;

cMAM: combined Moderate Acute Malnutrition;

cSAM: combined Severe Acute Malnutrition
