# Supplementary material for: Factors associated with acute malnutrition among children aged 6–59 months in Haiti, Burkina Faso and Madagascar: A pooled analysis
Source: PLoS One. 2022 Dec 12;17(12):e0278980. doi: 10.1371/journal.pone.0278980 (PMC9744306; doi:10.1371/journal.pone.0278980)
Supplement: S5 Table — For each indicator, several random effect mixed models were tested and compared to find the best model according to the AIC criterion. Different random-effect combinations were tested: country alone, cluster alone, cluster and country, and cluster nested in country. For Global Acute Malnutrition and Moderate Acute Malnutrition (WHZ, MUAC and indicators combined), the best model was the one with cluster nested in country. For Severe Acute Malnutrition, the best model was the one with cluster alone. Cluster: the smallest geographic units in each country. WHZ: Weight for Height Z-score; MUAC: Mid-upper Arm Circumference. cGAM: combined Global Acute Malnutrition; cMAM: combined Moderate Acute Malnutrition; cSAM: combined Severe Acute Malnutrition. Std. Dev.: Standard deviation. * Conditional modes of the random effects. (DOCX) [file pone.0278980.s008.docx]

**S5 Table. Random effects results from multivariate models**

|  |  | WHZ | | | MUAC | | | combined WHZ and MUAC | | |
| --- | --- | --- | --- | --- | --- | --- | --- | --- | --- | --- |
|  |  |  |  |  |  |  |  |  |  |  |
|  |  | WHZ < -2 | -3 ≤ WHZ < -2 | WHZ < -3 | MUAC < 125 | 115 ≤ MUAC < 125 | MUAC < 115 | cGAM | cMAM | cSAM |
| *Cluster : Country* | *Variance* | 0.5 | 0.3 | - | 25.7 | 1.4 | - | 0.5 | 0.3 | - |
|  | *Std.Dev.* | 0.7 | 0.6 | - | 5.1 | 1.2 | - | 0.7 | 0.5 | - |
|  |  |  |  |  |  |  |  |  |  |  |
| *Country* | *Variance* | 0.1 | 0.2 | - | 0.0 | 0.9 | - | 0.3 | 0.5 | - |
|  | *Std.Dev.* | 0.3 | 0.5 | - | 0.0 | 1.0 | - | 0.6 | 0.7 | - |
|  |  |  |  |  |  |  |  |  |  |  |
|  | (Intercept)* |  | | |  | | |  | | |
|  | *Madagascar* | 0.5 | 0.6 | - | 0.0 | 1.5 | - | 0.8 | 1.0 | - |
|  | *Haiti* | -0.2 | -0.3 | - | 0.0 | -0.5 | - | -0.4 | -0.6 | - |
|  | *Burkina Faso* | -0.02 | -0.01 | - | 0.0 | 0.2 | - | -0.1 | -0.1 | - |
| *Cluster* | *Variance* | - | - | 3.4 | - | - | - | - | - | 3.9 |
|  | *Std.Dev.* | - | - | 1.8 | - | - | - | - | - | 2.0 |

For each indicator, several random effect mixed models were tested and compared to find the best model according to the AIC criterion. Different random-effect combinations were tested: country alone, cluster alone, cluster and country, and cluster nested in country. For Global Acute Malnutrition and Moderate Acute Malnutrition (WHZ, MUAC and indicators combined), the best model was the one with cluster nested in country. For Severe Acute Malnutrition, the best model was the one with cluster alone.

Cluster: the smallest geographic units in each country

WHZ: Weight for Height Z-score; MUAC: Mid-upper Arm Circumference

cGAM: combined Global Acute Malnutrition; cMAM: combined Moderate Acute Malnutrition; cSAM: combined Severe Acute Malnutrition

Std. Dev.: Standard deviation

* Conditional modes of the random effects.
